# Clock Rooting Further Demonstrates that Guinea 2014 EBOV is a Member of the Zaïre Lineage

**DOI:** 10.1371/currents.outbreaks.c0e035c86d721668a6ad7353f7f6fe86

**Published:** 2014-06-16

**Authors:** Sébastien Calvignac-Spencer, Jakob M. Schulze, Franziska Zickmann, Bernhard Y. Renard

**Affiliations:** Robert Koch-Institute, Berlin, Germany; Robert Koch-Institute, Berlin, Germany; Robert Koch-Institute, Berlin, Germany; Robert Koch-Institute, Berlin, Germany

## Abstract

While initial phylogenetic analyses concluded to Guinea 2014 EBOV falling outside the Zaïre lineage (ZEBOV), a recent re-analysis of the same dataset by Dudas and Rambaut (2014) suggested that Guinea 2014 EBOV actually is ZEBOV. Under the same hypothesis as used by these authors (the molecular clock hypothesis), we reinforce their conclusion by providing a statistical assessment of the location of the root of the Zaïre lineage. Our analysis unambiguously supports Guinea 2014 EBOV as a member of the Zaïre lineage. In addition, we also show that some uncertainty exists so as to the location of the root of the genus Ebolavirus. We release the software we used for these re-analyses. RootAnnotator allows for the easy determination of branch root posterior probability from any posterior sample of clocked trees and is freely available at http://sourceforge.net/projects/rootannotator/.

## Introduction

Phylogenetic analyses are a very popular and powerful way to extract information from sequences. The end product is a tree-like graph intended to summarize the course of evolution. Sequence relatedness can be discussed on the basis of this graph but only on condition that it is assigned a direction for time. This is achieved by a process known as rooting, that turns a tree-like graph into a proper phylogenetic tree^1^ .

Dudas and Rambaut (2014) recently demonstrated that improper rooting can end up in supporting strikingly erroneous evolutionary scenarios, which can in turn mislead the formulation of important epidemiological hypotheses^2^ . These authors contradicted a preliminary report that identified Guinea 2014 Ebolavirus (EBOV) as a divergent lineage falling outside the Zaïre lineage (ZEBOV)^3^ , and suggested that Guinea 2014 EBOV instead nests within this lineage, i.e. is ZEBOV. Accordingly, Guinea 2014 EBOV is more likely to be the result of a fairly recent introduction of ZEBOV from Central Africa than a long-term endemic in West Africa.

The initial misplacement of Guinea 2014 EBOV was due to unnoticed long-branch attraction to the outgroup^4^ . As pointed out by Dudas and Rambaut (2014), this phenomenon made the long branch of Guinea 2014 EBOV drift towards the basis of the Zaïre clade as it was attracted to the other (very divergent) EBOV lineages included in the analyses (Bundibugyo, Taï Forest, Reston and Sudan)^2^ . To identify the location of the root within the Zaïre clade, Dudas and Rambaut (2014) excluded any outgroup and rooted the ingroup tree by minimizing the variance of root-to-tip distances (using Path-O-Gen, available at http://tree.bio.ed.ac.uk/software/pathogen/)^2^ . By doing so, they made the reasonable hypothesis that ZEBOV sequences evolve according to some kind of molecular clock and that the most likely root location minimizes rate variation across lineages.

This approach is very sensible. However, here we introduce an alternative method which is built under the same hypothesis but additionally allows for a quantitative assessment of the support for any root location. As an illustration, we apply this method to localize the root within the Zaïre clade. Using this same tool, we also investigate the position of the root within the genus *Ebolavirus*, whose deep branching order is controversial^14^^,^ 15 .

## Rationale

Over the last decade, one of the most fundamental developments in the field of phylogenetics was the introduction of models employing relaxed molecular clocks^5^ . These are clock models that recognize some degree of clocklikeness to the substitution process without going to the extreme of a single constant rate of evolution applied to the entire tree. This methodological leap considerably popularized phylogenetic analyses under clock models, which are commonly performed on one of the two leading platforms for Bayesian phylogenetics, BEAST^6^^,^ 7 and MrBayes^8^^,^ 9 ^,^ 10 . These platforms use Markov chain Monte Carlo (MCMC) samplers to approximate the (Bayesian) posterior distribution of all model parameters. Trees (i.e. topology and branch lengths) are among the parameters to estimate and MCMC samplers typically end up generating plausible sets of phylogenetic trees. When the model of evolution incorporates a clock model, MCMC samplers actually generate plausible sets of rooted trees. This can be viewed as generating a plausible set of tree-like graphs on the one hand and a plausible set of root locations on the other. Branch support is typically derived from posterior tree samples by counting the proportion of trees comprising the branch of interest (branch posterior probability). Following others^5^^,^ 11 ^,^ 12 , we propose to apply the same logic to derive root support. Here, the proportion of posterior trees for which the branch of interest wears the root would be taken as this branch root posterior probability (RPP).

## Software

Assessing branch RPP only requires parsing the posterior sample of trees, recording the branches wearing the root and their frequency. Due to the large number of trees, this cannot be done manually. As, to the best of our knowledge, no software is available for this purpose, we developed RootAnnotator, a user-friendly, portable software that collects information on root positions in posterior samples of trees and annotates a target tree with the according RPP (available from www.sourceforge.net/projects/rootannotator/). Among other options, the user can choose that the target tree is the maximum clade credibility (MCC) tree, which RootAnnotator will identify by running TreeAnnotator (distributed with BEAST)^6^ .

## Phylogenetic analysis

To investigate the position of the root within the Zaïre clade, we used the alignment of concatenated coding sequences published by Dudas and Rambaut (2014) (available at https://github.com/evogytis/ebolaGuinea2014) from which we removed all sequences that did not belong to the Zaïre lineage^2^ . This dataset therefore comprised 23 ZEBOV sequences and the three Guinea 2014 EBOV sequences. To assess the position of the root within the genus *Ebolavirus*, we also used an alignment of concatenated coding sequences derived from Dudas and Rambaut (2014)^2^ . This alignment included 5 sequences which were selected to represent the five recognized species in the genus.****

The first alignment was analyzed in BEAST using a GTR+Γ model of nucleotide substitution and assuming an uncorrelated relaxed clock (lognormal) which was tip-calibrated. We performed these analyses under the same three distinct demographic priors used by Dudas and Rambaut (2014) (constant population size, exponential growth and Bayesian skyride)^2^ . The second alignment was first analyzed in PhyML^13^ using a GTR+Γ model of nucleotide substitution. The resulting tree was analysed with Path-O-Gen which did not evidence any strong clocklike signal (a positive correlation of time and root-to-tip distances was only marginally supported; R^2^: 0.43). The second alignment was therefore analyzed in BEAST using a GTR+Γ model of nucleotide substitution and assuming an uncorrelated relaxed clock (lognormal) which was not tip-calibrated. We performed these analyses under two speciation priors (Yule and Birth-Death process).

Branch RPP were determined using RootAnnotator and plotted on the MCC tree**s**. When a branch appearing at least once as wearing the root in the posterior sample did not appear in the MCC tree, RootAnnotator was used to select a tree containing that branch so as to visualize their RPP (note that the RootAnnotator output also comprises a csv file comprising a list of all branches identified as wearing the root together with the associated RPP).

**Maximum clade credibility tree of concatenated coding sequences belonging to the Zaïre clade.**
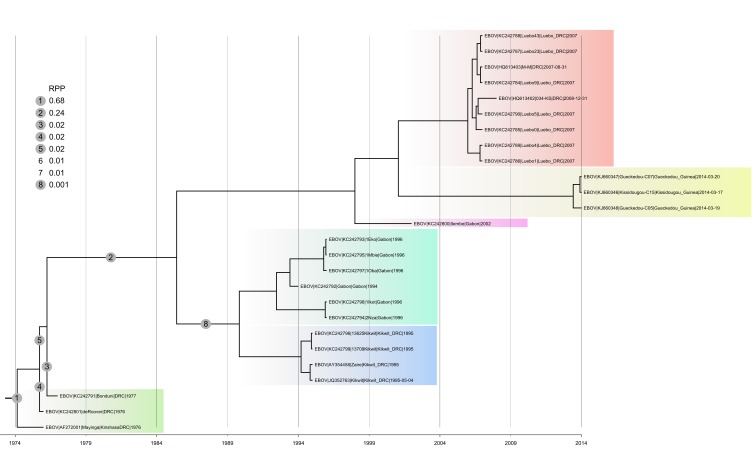
MCC tree and branch root posterior probabilities (RPP) derived from the analysis run under a constant population size model (the two other models ended up with generating very similar results) and an uncorrelated relaxed clock (lognormal). In the top left corner the complete list of branches that appeared at least once in the posterior tree sample and the according RPP. Note that two possible root locations (6 and 7) do not appear in the tree as the MCC tree did not comprise the corresponding branches. All internal branches linking coloured clades/groups received very good support (posterior probability: 1.00). The only exception was the branch defining the clade comprising Guinea 2014 EBOV and DRC 2007/2008 EBOV, which was only moderately supported (posterior probability comprised between 0.56 and 0.68).

## Results and discussion

For the Zaïre lineage, the posterior tree samples that we analyzed (one sample per demographic model) did not comprise a single tree whose root would be located on the branch leading to Guinea 2014 EBOV (Figure 1). Hence, under the assumption of a relaxed molecular clock it seems extremely unlikely that this virus falls outside the genetic diversity of the Zaïre lineage. The clock rooting approach implemented here therefore provides strong statistical support to the conclusion reached by Dudas and Rambaut (2014)^2^ . We also note that in our analyses the split of Guinea 2014 EBOV and the closest Central African EBOV was inferred to have taken place in 1999 (Bayesian skyride; 95% HPD interval 1996-2004) or 2001 (constant population size or exponential growth; 95% HPD interval 1996-2003), which comes very close to the GP-based estimates of Dudas and Rambaut (2002; 95% HPD interval 2000-2006)^2^ .

Depending on the demographic model, eight to nine root locations were identified within the Zaïre clade. Irrespective of the demographic model, the same two branches were always identified as receiving the two highest RPP. The external branch leading to the DRC 1976 ZEBOV strain (Mayinga) received RPP comprised between 0.62 and 0.69 whereas for the branch defining the bipartition [DRC 1976/1977 ZEBOV strains|other ZEBOV strains] RPP were between 0.21 and 0.28. These results mostly raise the question of the reciprocal monophyly of early DRC ZEBOV and all other ZEBOV strains (only supported by the second-to-best root location).

**Maximum clade credibility tree of concatenated coding sequences representing the 5 species of the genus Ebolavirus.**
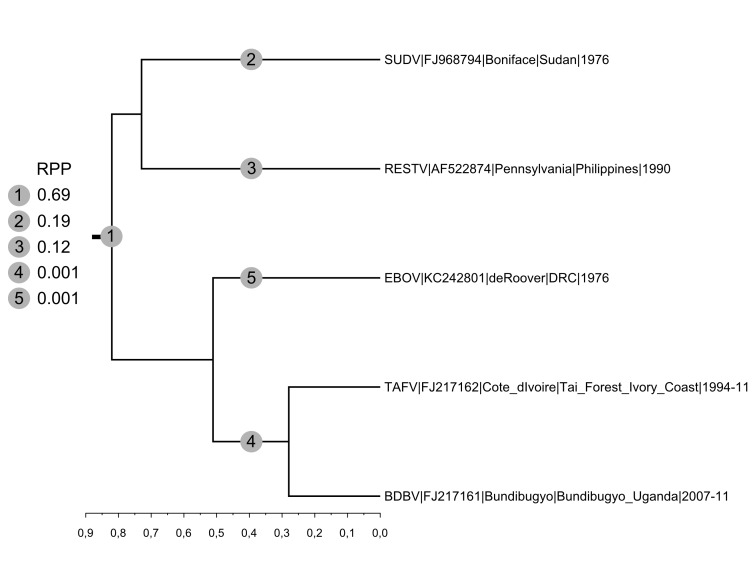
MCC tree and branch root posterior probabilities (RPP) derived from the analysis run under a Yule process (a Birth-Death process ended up with generating very similar results) and an uncorrelated relaxed clock (lognormal). The clock was not calibrated and the scale axis therefore is in substitution per site. RPP are reported in the list appearing at the left of the tree. All internal branches received very good support (posterior probability: 1.00).

We also applied the clock rooting approach to the genus *Ebolavirus*. Two recent articles whose analyses included outgroup sequences (Marburg virus and Lloviu virus) agreed on the monophyly of Bundibugyo, Taï Forest and Zaïre ebolaviruses but supported different sisterships of this clade with Reston and Sudan ebolaviruses. In the phylogeny produced by Lauber and Gorbalenya (2012) Reston ebolavirus was in sistership with the clade comprising Zaïre ebolavirus^15^ while in the phylogeny by Carroll et al. (2013) Sudan and Reston ebolaviruses formed a sister clade to the clade comprising Zaïre ebolavirus^14^ .

Under both speciation priors we tested, five root locations were identified and among these three gathered >0.99 RPP (Figure 2). The branch defining the bipartition [Sudan, Reston|Bundibugyo, Taï Forest, Zaïre] received RPP 0.69 and 0.68 (Yule and Birth-Death process, respectively), the external branch leading to Sudan ebolavirus RPP 0.19 and 0.18 and the external branch leading to Reston ebolavirus RPP 0.12 and 0.13. Therefore, while the hypothesis put forward by Carroll et al. (2013) gets more probabilistic support^14^ , our analyses underlines significant uncertainty and the existence of a third plausible root position. As Reston ebolavirus is the only Asian ebolavirus (all other ebolaviruses are African), we note here that this new rooting scenario would imply a geographical partitioning of the genetic diversity within the genus *Ebolavirus*.****

In our view, these examples highlight the unique ability of clock rooting to capture uncertainty so as to root location. With RootAnnotator it is now easily possible to establish short lists of plausible roots warranting further examination^12^ , even where no obvious candidate roots exist (e.g. in the absence of appropriate outgroups). We hope that this tool will provide the many biologists using Bayesian phylogenetics under clock models with a rationale to turn even more tree-like graphs into phylogenetic trees.

## Competing interests

The authors have declared that no competing interests exist.
